# Aldehyde Dehydrogenase: An Off-Label Marker of Endothelial Activation in Oral Squamous Cell Carcinoma

**DOI:** 10.7759/cureus.41596

**Published:** 2023-07-09

**Authors:** Dimitrios Andreadis, Vasileios Zisis, Pinelopi Anastasiadou, Lefteris Anagnostou, Konstantinos Paraskevopoulos, Athanasios Poulopoulos

**Affiliations:** 1 Oral Medicine/Pathology, Aristotle University of Thessaloniki, Thessaloniki, GRC; 2 Oral Medicine, Aristotle University of Thessaloniki, Thessaloniki, GRC; 3 Oral and Maxillofacial Surgery, General Hospital of Thessaloniki "George Papanikolaou", Thessaloniki, GRC

**Keywords:** vascular endothelial cells, cancer stem cell biomarker, oral leukoplakia, aldehyde dehydrogenase, oral cavity squamous cell carcinoma, cancer stem cells

## Abstract

Introduction: The vascular endothelial (VE) expression of aldehyde dehydrogenase (ALDH) 1/2 family in oral leukoplakia (OL) and oral squamous cell carcinoma (OSCC) cases has not been studied so far. The aim of this study was to illustrate the “off-label” endothelial expression of cancer stem cell (CSC) biomarker, ALDH1/2, adjacent to oral potentially malignant and malignant lesions in order to shed some light on the mechanisms leading to oral carcinogenesis.

Materials and methods: The expression of CSC protein-biomarker ALDH1/2 was detected through immunohistochemistry (IHC) in 30 paraffin-embedded samples of OL and 21 samples of OSCC compared to five samples of normal oral mucosa. Statistical analysis was done using SPSS, Pearson Chi-square, and Fischer’s exact test. The significance level was set at 0.05 (p≤ 0.05).

Results: In oral mucosal vessels, ALDH1/2 was not expressed. It was expressed significantly more in the vessels of OSCCs compared to the OLs (Fisher’s exact test, p-value= 0,001). However, when endothelial expression of ALDH1/2 in the vasculature of OLs was compared with that of the normal oral mucosa, no significant change was noticed (Fisher’s exact test, p-value=1.000).

Discussion: The IHC VE expression of ALDH1/2 in OSCC vasculature but not in OL indicates a possible significantly stronger activation of endothelial cells during carcinogenesis, which could be an indicator of the role of inflammation in the development of field cancerization and of prognostic value for (vascular/lymphatic) metastasis.

## Introduction

The term “oral potentially malignant disorder" (OPMD) is attributed to oral mucosal disorders/lesions that exhibit an increased risk for malignant transformation compared to healthy mucosa [1]. The most common OPMD is oral leukoplakia (OL) [2]. Malignant conversion of OL results in oral squamous cell carcinoma (OSCC), arising from cells of the stratified squamous epithelium. Its biological behavior and other clinical and microscopic/molecular parameters affect therapeutic procedures and prognosis [3].

Oral carcinogenesis, according to the cancer stem cell (CSC) theory, is caused by a particular population of CSCs. CSCs are at the top of the cancer cell hierarchy [4]. CSC biomarkers are used to detect subpopulations of cancer cells with “stemness” (characteristics of stem cells) and are subdivided into markers of embryonic stem cells and markers showing the presence of stem cell traits in certain cells (stemness). Aldehyde dehydrogenases (ALDH) are a group of enzymes that catalyze the oxidation of aldehydes and function as stemness markers [5]. There is expression of ALDH in vascular endothelial (VE) cells in the tumor blood vessels of in vivo mouse models of oral carcinoma while there is no expression in normal blood vessels [6]. Angiogenesis is crucial for the process of tumorigenesis and metastasis [7]. The most important angiogenic factors are the three peptide families of VE growth factor (VEGF), fibroblast growth factor (FGF), and platelet-derived growth factor (PDGF) [7].

The aim of this study is to illustrate the CSC biomarker ALDH1/2 expression in the VE cells adjacent to potentially malignant and malignant lesions.

## Materials and methods

This study examines the immunohistochemical expression of the anti-ALDH1/2 index (sc-166362; Santa Cruz Biotechnology, Dallas, Texas, United States) in tissue samples from histopathologically confirmed cases of OL of all degrees of dysplasia and OLCC of all degrees of differentiation in comparison to five cases of normal mucosa (neighboring healthy epithelium from reactive hyperplasia, e.g. fibroma). The full laboratory process was conducted at the Laboratory of Stomatology, Dental School of Aristotle University of Thessaloniki, Greece. The study adhered to international and national research methodological standards and was authorized by the Ethics Committee of the Dental School of Aristotle University of Thessaloniki, Greece (approval number: 8/03.07.2019). 

The samples were put in formaldehyde (10%) immediately after their surgical removal and were later preserved in paraffin. The study included 30 samples of OL of all degrees of dysplasia, 21 samples of OSCC of all degrees of differentiation, and five samples of histologically normal oral epithelium. The cases/samples were divided into three categories: OL, OSCC, and Normal. OL and OSCC were further subdivided into two subcategories each: Moderately and Severely Dysplastic OL, Mildly Dysplastic and Non-dysplastic OL, Moderately and Poorly Differentiated OSCC, and Well-Differentiated OSCC (Table 1).

**Table 1:** Demographical and clinical manifestation details of the patients from which derived the tissue samples. M: male; F: female

	CATEGORY & SUBCATEGORY	LOCATION	GENDER	AGE
1	Leukoplakia/Moderately & Severely Dysplastic	Tongue	F	44
2	Leukoplakia/Moderately & Severely Dysplastic	Tongue	F	60
3	Leukoplakia/Moderately & Severely Dysplastic	Tongue	M	58
4	Leukoplakia/Moderately & Severely Dysplastic	Tongue	F	67
5	Leukoplakia/Moderately & Severely Dysplastic	Tongue	F	62
6	Leukoplakia/Moderately & Severely Dysplastic	Cheek	M	66
7	Leukoplakia/Moderately & Severely Dysplastic	Cheek	M	67
8	Leukoplakia/Moderately & Severely Dysplastic	Tongue	M	43
9	Leukoplakia/Moderately & Severely Dysplastic	Buccogingival Sulcus	F	75
10	Leukoplakia/Moderately & Severely Dysplastic	Tongue	M	50
11	Leukoplakia/Moderately & Severely Dysplastic	Buccogingival Sulcus	M	59
12	Leukoplakia/Moderately & Severely Dysplastic	Tongue	M	75
13	Leukoplakia/Moderately & Severely Dysplastic	Tongue	M	64
14	Leukoplakia/Moderately & Severely Dysplastic	Tongue	M	45
15	Leukoplakia/Moderately & Severely Dysplastic	Palate	M	72
16	Leukoplakia/Moderately & Severely Dysplastic	Tongue	F	84
17	Leukoplakia/Mildly Dysplastic & Non Dysplastic	Tongue	F	61
18	Leukoplakia/Mildly Dysplastic & Non Dysplastic	Lip	F	38
19	Leukoplakia/Mildly Dysplastic & Non Dysplastic	Tongue	M	46
20	Leukoplakia/Mildly Dysplastic & Non Dysplastic	Gingiva	F	12
21	Leukoplakia/Mildly Dysplastic & Non Dysplastic	Tongue	F	45
22	Leukoplakia/Mildly Dysplastic & Non Dysplastic	Tongue	M	67
23	Leukoplakia/Mildly Dysplastic & Non Dysplastic	Cheek	F	60
24	Leukoplakia/Mildly Dysplastic & Non Dysplastic	Tongue	F	68
25	Leukoplakia/Mildly Dysplastic & Non Dysplastic	Tongue	M	69
26	Leukoplakia/Mildly Dysplastic & Non Dysplastic	Tongue	F	68
27	Leukoplakia/Mildly Dysplastic & Non Dysplastic	Cheek	F	58
28	Leukoplakia/Mildly Dysplastic & Non Dysplastic	Cheek	F	61
29	Leukoplakia/Mildly Dysplastic & Non Dysplastic	Cheek	M	75
30	Leukoplakia/Mildly Dysplastic & Non Dysplastic	Corner of the mouth	M	37
31	OSCC/Poorly & Moderately Differentiated	Tongue	F	47
32	OSCC/Poorly & Moderately Differentiated	Tongue	F	53
33	OSCC/Poorly & Moderately Differentiated	Cheek	M	45
34	OSCC/Poorly & Moderately Differentiated	Tongue	F	77
35	OSCC/Poorly & Moderately Differentiated	Cheek	M	66
36	OSCC/Poorly & Moderately Differentiated	Cheek	M	61
37	OSCC/Poorly & Moderately Differentiated	Mouthfloor	F	76
38	OSCC/Poorly & Moderately Differentiated	Tongue	M	75
39	OSCC/Poorly & Moderately Differentiated	Tongue	F	79
40	OSCC/Poorly & Moderately Differentiated	Cheek	F	78
41	OSCC/Poorly & Moderately Differentiated	Tongue	F	72
42	OSCC/Poorly & Moderately Differentiated	Tongue	M	52
43	OSCC/Poorly & Moderately Differentiated	Tongue	M	60
44	OSCC/Poorly & Moderately Differentiated	Tongue	F	77
45	OSCC/Poorly & Moderately Differentiated	Tongue	F	80
46	OSCC/Poorly & Moderately Differentiated	Mouthfloor	F	82
47	OSCC/Well Differentiated	LIP	M	58
48	OSCC/Well Differentiated	LIP	M	77
49	OSCC/Well Differentiated	Tongue	F	73
50	OSCC/Well Differentiated	Tongue	F	67
51	OSCC/Well Differentiated	Tongue	M	43
52	Normal	Tongue	F	49
53	Normal	Cheek	M	81
54	Normal	Cheek	F	59
55	Normal	Tongue	M	69
56	Normal	Tongue	F	72

For the IHC technique application, the incisions were mounted on slides. The material was processed using anti-ALDH1/2 antibody (sc-166362) at a dilution of 1:100 using the Dako EnVision FLEX+ Visualization Systems (Agilent Technologies, Inc., Santa Clara, California, United States). Specifically, the staining process included antigen recovery, the application of primary antibody, the application of the EnVision™ FLEX DAB+ Substrate Chromogen System (Agilent Technologies, Inc.), the chromogenic agent application (Dako Dab Envision) (Chromogen), and finally the application of hematoxylin.

The incision was then affixed to the mounting plate and coated to protect and preserve the preparation over time. The evaluation of IHC staining was performed by microscopically examining the incisions in order to observe and record the results. Two observers (DA and VZ) evaluated the staining. The process was blinded and during the evaluation, only a five-digit code, attributed to each tissue sample, was known to the observers. The vascular staining was evaluated either as positive or negative. The IHC staining was evaluated as positive when the cytoplasm and/or the membrane of at least one endothelial cell was depicted in brown in an area of the same size in all samples. 

Statistical analysis was performed using IBM SPSS Statistics for Windows, Version 25.0 (Released 2017; IBM Corp., Armonk, New York, United States). Pearson Chi-square test and Fisher’s Exact test were used depending on the sample size. The significance level was set at 0.05 (p≤ 0.05).

## Results

**Figure 1:**
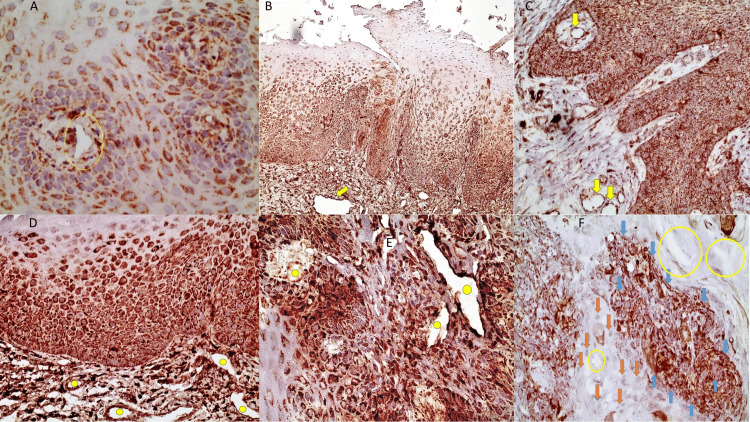
Characteristic immunohistochemical images of both positive and negative vascular endothelial staining in normal oral epithelium, OL, and OSCC. (A) Lack of staining in endothelial cells in normal oral epithelium (yellow circle) (X40); (B) Strong ALDH expression in endothelial cells of large vessels (Moderately to Severely Dysplasic OL) (yellow arrow) (X10); (C) ALDH positive endothelial cells in vessels in OL transformed to invasive well-differentiated OSCC (yellow arrows) (X20); (D) Strong ALDH expression of endothelial cells in small vessels of OL (Moderately to Severely Dysplastic OL epithelium) (yellow spots) (X20); (E) Strong endothelial ALDH positivity in poorly differentiated OSCC (yellow spots) (X40). There is also strong positivity of neoplastic epithelial cells; (F) Increased membrane and cytoplasmic staining in moderately-differentiated OSCC (blue arrows) (X40). The stromal fibroblasts are negative (orange arrows). In this case, the endothelial cells of the vessels were negative for ALDH1/2 (yellow circles). OL: oral leukoplakia; ALDH: Aldehyde dehydrogenase; OSCC: oral squamous cell carcinoma

Despite the IHC expression of ALDH1/2 in the normal epithelium (the pattern of positive epithelial expression includes the cytoplasmic and membranous staining of individual spindle cells and of the basal cell layer in contrast to the negative endothelial cells underlying the epithelium), the endothelial staining of ALDH was negative in the vessels of the oral mucosa (Figure 1A). The endothelial staining was also negative in cases of mildly dysplastic and non-dysplastic OL. In contrast, in five moderately and severely dysplastic OL samples (out of 30 OL samples), ALDH1/2 was strongly expressed in the endothelial cells of vessels adjacent to the superficial dysplastic epithelium (the pattern of positive epithelial expression in moderately and severely dysplastic OL samples includes the cytoplasmic and membranous staining of two-thirds of the epithelium whereas the pattern of positive VE expression includes the cytoplasmic and membranous staining of the endothelial cells, underlying the OL lesion) (Figure 1 B-D).

However, no statistically significant difference was noticed compared to the normal oral mucosa (Fisher’s exact test, p-value= 1.000). Interestingly, this partial positivity in worse cases of OL was dramatically increased in endothelial cells of 13 out of 21 OSCC samples, irrespective of the grade of differentiation but with statistical significance compared to OL (Fisher’s exact test, p-value= 0,001) (the pattern of positive epithelial expression in OSCC cases includes the cytoplasmic and membranous staining of more than two-thirds of the epithelium whereas the pattern of positive VE expression includes the cytoplasmic and membranous staining of the endothelial cells adjacent to the cancerous foci and the overlying dysplastic epithelium) (Figure 1 E-F). The positivity was also statistically significantly higher in OSCC than in normal cases (Fisher’s exact test, p-value=0,039) (Table 2).

**Table 2:** Statistical analysis of the vascular endothelial expression of ALDH in normal, OL, and OSCC samples. OL: oral leukoplakia; ALDH: Aldehyde dehydrogenase; OSCC: oral squamous cell carcinoma

Statistical comparison	Normal- OL	OL- OSCC	Normal- OSCC
Similar levels of expression	Fisher’s exact test, p value= 1.000		
Different levels of expression		Fisher’s exact test, p-value= 0,001 (OSCC > OL)	Fisher’s exact test, p-value= 0,039 (OSCC > Normal)

The aforementioned findings imply that the expression of ALDH is detected in higher levels in general in cancer. The positive staining of ALDH1/2 of the endothelial cells of the vessels adjacent to cancerous foci in OSCC cases was noticed. The demographical and clinical manifestation details of the patients from which the positively stained tissue samples were derived are given in Table 3.

**Table 3:** Demographical and clinical details of the 18 patients from whom the positively-stained samples were derived OL: oral leukoplakia; OSCC: oral squamous cell carcinoma

DIAGNOSIS	LOCATION	GENDER	AGE
Moderately Dysplastic OL	Tongue	Male	58
Severely Dysplastic OL	Tongue	Male	43
Severely Dysplastic OL	Mucobuccal fold	Female	75
Severely Dysplastic OL	Tongue	Male	45
Severely Dysplastic OL	Palate	Male	72
Well-Differentiated OSCC	Lip	Male	58
Well-Differentiated OSCC	Lip	Male	77
Well-Differentiated OSCC	Tongue	Female	67
Moderately Differentiated OSCC	Cheek	Male	61
Moderately Differentiated OSCC	Tongue	Male	75
Moderately Differentiated OSCC	Tongue	Female	79
Moderately Differentiated OSCC	Tongue	Female	72
Moderately Differentiated OSCC	Tongue	Female	47
Moderately Differentiated OSCC	Cheek	Male	45
Poorly Differentiated OSCC	Tongue	Female	77
Poorly Differentiated OSCC	Tongue	Male	52
Poorly Differentiated OSCC	Tongue	Female	77
Poorly Differentiated OSCC	Mouthfloor	Female	82

## Discussion

VE staining of ALDH has been reported only in tissues of kidney cancer (specifically renal cell carcinoma, a well-known angiogenic tumor) where double immunofluorescence staining of the frozen sections of human renal tumors and normal kidney tissues was performed using anti-ALDH antibody, and the ALDH staining was proven to be negative in normal blood vessels, but was strongly positive in tumor blood vessels [6]. Additionally, in in vivo mouse models of oral carcinoma, double immunofluorescence staining of oral carcinoma xenografts in mice was carried out using anti-ALDH antibody, and VE staining of ALDH has been reported in VE cells of tumor blood vessels within tumor foci [6]. However, ALDH was expressed in the tumor blood vessels of oral carcinoma xenografts, indicating that oral carcinoma contains ALDH high endothelial cells. ALDH was hardly expressed in normal blood vessels in vivo [6]. Additionally, the pattern of ALDH expression in tumor blood vessels was heterogeneous, which suggests that stem-like endothelial cells are present in tumor blood vessels in vivo [6].

Interestingly, endothelial cells positive for ALDH showed drug resistance to 5-FU in vitro and in vivo and manifested higher levels of aneuploidy [8]. Therefore, ALDH may be applied as a prognostic marker for drug resistance and genetic aberrations. High ALDH activity was found in a subset of human mesenchymal stromal cells with vascular regenerative potential [9], suggesting a preexisting cell capacity for a pro-angiogenic secretory role, which either initiates or mediates the angiogenesis taking place, close to dysplasia or cancer. Tumor endothelial cells (TECs) are generally different from their normal counterparts due to different gene expression [10,11] The chemokine receptor CXCR7 is upregulated in TECs and therefore constitutes a novel marker [12]. Lysyl oxidase is also upregulated in TECs and its knockdown inhibited cell migration, and thus may be applied as a biomarker for angiogenesis [13]. Prostacyclin receptor mediates re-endothelialization and angiogenesis, constituting another novel biomarker for neoangiogenesis [14]. Biglycan is also upregulated in TECs and its knockdown inhibited cell migration, and thus may be applied as a biomarker for angiogenesis [15].

TECs may acquire cytogenetic abnormalities while in the tumor microenvironment and these cytogenetic alterations in tumor vessels of carcinoma may play a significant role in modifying tumor-stromal interactions [16,17]. Finally, TECs from high metastatic tumors have a more pro-angiogenic phenotype than those from low metastatic tumors [18]. In our study, we found (for the first time) a strong positivity of ALDH in OSCC tissues (mainly) and in the advanced stages of OL. Further studies in larger samples of patients with a wider variety of ALDH biomarkers may shed some light on the possible role of endothelial expression of ALDH in tumor neo-angiogenesis (blood or lymphatic) and possible relevant therapeutic implications. It may be the case, based on the findings of the aforementioned studies, that specific gene expression alters the TECs which in turn attract cancer cells on a locoregional level. The next step may be the epithelial to mesenchymal transition of cancer cells, enabling the migration through the adjacent vessels, and thus resulting in metastatic lymphadenopathy and distant metastasis. Additionally, the positive VE staining in severe OL may indicate that the microenvironment of dysplastic OL interacts with the underlying stroma. Since FGF, especially FGF-2, plays a significant angiogenic role [7], fibroblasts may also mediate the interaction between dysplastic epithelial cells and endothelial cells. Inflammation may also influence angiogenesis through the function of transforming growth factor beta (TGF-β), interferons (IFNs), TNF-a, and interleukins [7]. TGF-β, IFNs, and TNF-a still play a controversial role but interleukins on the other hand have proven to be an important pro-angiogenic factor [7]. A reverse scenario may also be assumed where inflammation initiates the stroma alterations leading to VE alterations leading eventually to epithelial dysplasia and carcinogenesis. Finally, ALDH is implicated in initiating the process of field cancerization, which is a biological process in which large areas of cells at a tissue surface or within an organ are affected by carcinogenic alterations [19]. Also, ALDH1 activity is correlated with poor clinical prognosis and higher recurrence rates [20].

The limitations of the study include the lack of quantitative assessment, the lack of TNM (tumor, nodes, and metastases) classification and five-year survival rate of the OSCC cases, as well as the lack of information regarding the course of the disease in general, its therapeutic approach, and the malignant transformation rate of the OL cases involved.

## Conclusions

The statistically significant high IHC VE expression of ALDH1/2 in OSCC than in OL and normal oral mucosa vasculature indicates a possible significantly stronger activation of endothelial cells during carcinogenesis that could be an indicator for the role of inflammation in the development of field cancerization and of prognostic value for (vascular/lymphatic) metastasis. Further studies in more patients and with additional information regarding TNM classification, the course of the disease, and the malignant transformation rate of OL may enhance the clinical importance of our findings.
